# Serum neurofilament light chain (sNFL) as a predictive biomarker for Chemotherapy-induced peripheral neurotoxicity (CIPN): consideration of current evidence, validation steps, and barriers to CIPN biomarker implementation in clinical practice—a narrative review

**DOI:** 10.1007/s00520-026-10690-2

**Published:** 2026-05-07

**Authors:** Paola Alberti, Sara Faithfull, Andreas A. Argyriou, David Balayssac, Daniel L. Hertz, Maryam Lustberg, Susanna B. Park, Tiffany Li, Mary Tanay, Terri Jabaley, Lise Worthen-Chaudhari, Ellen M. L. Smith

**Affiliations:** 1 School of Medicine & Surgery, University of Milano-Bicocca, Via Cadore 48, 20900 Monza, Italy; 2 Discipline of Radiation Therapy, School of Medicine, Trinity College Dublin, The University of Dublin, Dublin, Ireland; 3 Department of Neurology, “Agios Andreas” General Hospital of Patras, Kalavriton 37 Str, 26335 Patras, Greece; 4 Direction de la Recherche Clinique et de l’Innovation (DRCI), CHU Clermont-Ferrand (Clermont-Ferrand University Hospital), Clermont-Ferrand, France; 5 College of Pharmacy, University of Michigan, 428 Church St, Ann Arbor, MI 48109-1065 USA; 6 Yale School of Medicine, 333 Cedar St, New Haven, CT 06510 USA; 7 Faculty of Medicine and Health, School of Medical Sciences and Brain and Mind Centre, University of Sydney, Camperdown, NSW 2050 Australia; 8 Berkshire Cancer Centre, Royal Berkshire NHS Trust, Reading, UK; 9 Dana-Farber Cancer Institute, 450 Brookline Avenue, BP 1140L, Boston, MA 02215 USA; 10 Department of Physical Medicine and Rehabilitation, Dodd Rehabilitation Hospital, The Ohio State University College of Medicine, Colombus, OH 43210 USA; 11 Department of Acute, Chronic & Continuing Care, The University of Alabama at Birmingham School of Nursing, 2101 Magnolia Avenue, Room 225, Birmingham, AL 35205 USA; 12 NEURO-DOL, Université Clermont Auvergne, INSERM, UMR 1107 / U1107, Clermont-Ferrand, France

**Keywords:** Biomarkers, CIPN, Quality of life, Cancer survivors, sNfL, NfL

## Abstract

**Purpose:**

Several anticancer drugs can cause chemotherapy-induced peripheral neurotoxicity (CIPN) in approximately 70% of patients; of these, 30% continue to have chronic symptoms hampering quality of life. As more people live with and beyond cancer therapy, it is becoming increasingly important for clinicians to assess those at higher risk of CIPN and provide care for managing CIPN severity. Biomarkers could be appropriate in this regard. However, an initial step towards biomarker-informed care is to advance scientific knowledge regarding the potential of current biomarkers such as neurofilament light chain (NfL). Here, outcomes of a CIPN biomarker workshop and discussions among clinical and scientific experts held during the MASCC 2024 annual conference are reported. The aims of this work are to (1) identify knowledge gaps regarding the potential for serum NfL (sNfL) as a predictive CIPN biomarker, (2) provide guidance for future biomarker research, and (3) explore whether sNfL is ready for use in clinical practice.

**Methods:**

Consensus of the experts was gained using a Normative Group Technique. A two-stage iterative approach was used: Stage 1 involved a literature review and an in-person workshop; Stage 2 involved a virtual zoom event to confirm understanding and priorities following the workshop.

**Results:**

Eleven studies identified that increases in serum NfL after receiving 2–3 chemotherapy doses are associated with CIPN severity post treatment. Despite preliminary evidence suggesting positive correlations among sNfL and CIPN objective and subjective measures, wide variation in sNfL levels, the prescribed neurotoxic chemotherapy agent, dose and schedule, and symptom assessment time points make it difficult to use sNfL as a predictor of CIPN severity.

**Conclusions:**

While there is potential for sNfL to be a useful biomarker, larger scale research exploring the predictive value is needed before sNfL can be used to inform clinical decision-making. Identifying links with validated clinical assessments could be utilized to enhance biomarker accuracy and to address the multiple pathological mechanisms of neurotoxicity that occur from the differing chemotherapies. Moreover, workshop participants identified significant barriers to biomarker implementation (e.g. costs, long processing time, and unknown cut off points).

## Introduction

Chemotherapy induced peripheral neurotoxicity (CIPN) is characterized by a dose-dependent sensory neuropathy/neuronopathy. Seventy percent of patients experience symptoms in the first month of treatment; symptoms persist in 60% of patients at 3 months, with 30% continuing to have symptoms 6 months or longer after chemotherapy is discontinued [1]. Rates of CIPN vary widely across treatment types; higher rates are reported by those receiving platinum-based drugs, vinca alkaloids, and taxanes. CIPN manifests itself as sensory deficit mainly in the hands and feet, with sensory loss, numbness, neuropathic pain, thermal sensitivity, and fine motor dysfunction [2] impacting walking and increasing risk of falls [3, 4]. Long-term CIPN symptoms cause substantial disability and impact cancer survivors’ quality of life [5–7].

At present, there are no evidence-based interventions that prevent or reverse CIPN. Current clinical guidelines by the American Society of Clinical Oncology (ASCO) recommend that clinicians assess the appropriateness of dose reductions, dose delays, and substitutions in patients who develop intolerable CIPN during therapy [8]. Thus, there is a need to identify those at increased risk of developing severe and long-term CIPN earlier during chemotherapy to mitigate symptoms through chemotherapy treatment alterations [9]. Such treatment changes should be personalized, based on objective and subjective assessment, but also be balanced with the need to maximize treatment efficacy [10]. Although the risk of severe CIPN is typically related to the agent and cumulative dose, there remains substantial interindividual variation in CIPN development. This is likely due to variation in demographics, comorbidities, and genetic background, which influence CIPN risk. This variance may also reflect the multiple pathophysiological mechanisms involved in the development of peripheral neurotoxicity and the variation across chemotherapy drugs, which makes personal risk factors difficult to operationalize in risk prediction algorithms that can inform clinical practice.

Clinical implementation of a routine biological biomarker of CIPN could change the nature of the decision-making for dose reductions [11, 12]. The National Institute of Health (NIH) biomarker toolkit defines biomarkers as “*characteristics that can be objectively measured and used as an indicator of normal biological processes, disease processes, or pharmacologic response to a therapy*”. Biomarkers can be used for a range of purposes: prognostic in identifying the likelihood of an outcome in patients with the condition, or predictive through comparisons of those with and without the condition [13]. A clear understanding of the molecular pathways for CIPN development is helpful to define an appropriate CIPN biomarker that can predict clinical outcomes [11, 14]. Pre-clinical studies have identified multiple pathophysiological pathways which lead to axonal degeneration in CIPN models, supporting identification of biomarkers based on a sound biological rationale [14, 15]. There is a rapidly emerging commercial market of biomarkers for neurological illness [16] with several reviews of blood molecular biomarkers that may be useful for CIPN objective assessment and risk stratification [17–20]. Informed by these narrative reviews, researchers and clinicians should collaborate to advance our understanding of barriers and facilitators to using serum neurofilament light chain levels (sNfL) as a CIPN biomarker and identify gaps in our knowledge that may impact potential validation and adoption to guide future research.

While there are numerous biomarkers that may have utility to predict CIPN (e.g. omics, kinetics, genetics, vitamin D deficiency), biomarker workshop participants focused on a serum protein with significant potential for use in point-of-care clinical settings. Neurofilament light chain (NfL), a protein derived from the neuronal cytoskeleton, has been identified as a potential biomarker for CIPN and has been recommended as a biomarker in many neurological disorders. Peripheral nerve damage therefore results in release of NfL which can be detected in cerebral spinal fluid and blood serum [14]. NfL is a non-specific biomarker because increased values are seen across multiple Central Nervous System (CNS) or Peripheral Nervous System (PNS) disorders. Thus, NfL is not specific to CIPN, but is probably sensitive to CIPN occurrence [14, 15]. NfL emerged as a potential target of interest in 2016 for neurological disorders following the implementation of Single Molecule Array (SIMOA) technique with ultra-sensitive immunoassays that enable the measurement of biomarkers at lower levels. sNfL was first tested in CIPN animal models [15, 21] paving the way for further testing in clinical cohorts.

This manuscript is based on the multidisciplinary group discussions that took place during the MASCC 2024 annual conference (Stage 1) combined with a review of the literature and a virtual zoom meeting to confirm understanding and priorities for those involved in the workshop (Stage 2). Our aim was to inform healthcare professionals and researchers regarding the future potential of sNfL to predict onset and severity during and after anticancer therapy.

### Method

We used a Nominal Consensus Group Technique (NGT) [22], a method that is conducted face to face and is used when there is a paucity of evidence in the area [23]. We applied a two-stage iterative approach: Stage 1 involved a literature review and an in-person workshop; Stage 2 involved a virtual zoom event to confirm understanding and priorities following the workshop. Specifically, the NGT method has several key steps designed so that participants can share and compare experiences and reach consensus. We adapted the NGT method [24] by applying a two-stage iterative approach: Stage 1 involved a literature review for idea generation, presentations, round-robin feedback of ideas, and smaller group discussions providing clarification, all within an in-person workshop; Stage 2 involved a virtual zoom event to confirm understanding, rank ideas, and identify priorities following the workshop.

Specifically, concerning strengths and weaknesses, the NGT differs from other consensus techniques, for example the Delphi survey or RAND/UCLA, by employing an in-person meeting of between 8 and 20 participants. This is considered a strength, as it not only allows “discordant” views on the topic, but it also affords the opportunity to explore differing opinions and reasons for these. A weakness is that consensus exercises are susceptible to potential biases, both during the development phase but also influenced by the number and experiences of the participants involved. There is also the potential for dominant participants to unduly influence groups’ decision-making. These limitations this was mitigated by facilitators and clear structural procedures within the workshop.

A search strategy was developed to enable a structured search of published clinical trials and prospective studies using key words and MeSH terms, which included “chemotherapy induced peripheral neuropathy” “chemotherapy neurotoxicity”, “biomarkers”, “serum neurofilament light”, “NfL biomarker”, and “serum NfL levels”. Google Scholar and PubMed were used to search original research articles between 2014 and 2024. Reference lists were also searched. The Planning Committee initially reviewed the identified NfL literature, which in turn informed the focus of the workshop.

Workshop participants included 14 international and multidisciplinary representatives (basic scientist, clinical pharmacologist, neuroscientists, neurologists, oncologists, nurse scientists, a rehabilitation specialist, and a biomarker industry representative). Each participant was invited based on their clinical and/or scientific expertise regarding CIPN clinical manifestations, measurement, biomarkers, and management. Workshop participants were provided with sentinel publications [25, 26] to review in advance of the meeting.

Upon meeting for the in-person workshop, experts gave the following brief presentations: (1) General Introduction to Biomarkers, (2) Overview of CIPN Biomarkers, (3) Most Promising Biomarkers with High Potential for Clinical Application, and (4) Roadmap of Current and Future Pathways for CIPN Biomarker Tests in Clinical Settings. Following these presentations, participants were assigned to one of three distinct working groups according to their expertise, and each group was asked to respond to a single question that was relevant to their assigned topic area. Participants were aware that their responses would be used to form the basis for a consensus statement and publication. The findings from the workshop were incorporated in a draft report and circulated to those in the workshop for feedback. This was followed by a virtual event to rank priorities and develop consensus statements.

The workshop groups focused their discussion on three key questions identified by the Planning Committee.

1. What steps are needed to validate that a promising sNfL biomarker can predict CIPN clinical outcome?
2. What evidence is needed to demonstrate that sNfL-guided treatment decisions will improve CIPN clinical outcome?
3. What are the barriers to and facilitators of implementing sNfL biomarker collection into clinical practice?

The Planning Committee plus selected workshop participants (focus group leaders) reviewed the NGT results and relevant literature, and some served as manuscript coauthors. Workshop discussions are summarized below, in conjunction with the literature. It is to be noted that this was a consensus document developed with permission of the workshop participants with consent for publication prior to attending and therefore ethical approval was not deemed mandatory.

## Results

Pre-clinical CIPN biomarker studies were identified in the literature but were excluded in the evidence synthesis. The literature search identified 11 clinical studies, which are summarised in Table 1 with relevant study details. The sNfL studies were mainly conducted in women with breast cancer [17, 27–31]. There was one study conducted with patients treated for non-small cell lung cancer [32], two studies including patients treated for colorectal cancer [31, 33], and two studies enrolled women treated for gynaecological cancers [34, 35]. Ten of the studies explored the use of paclitaxel within multi-agent chemotherapy regimens and two focused on oxaliplatin [31, 33] and one on bortezomib [36]. Sample sizes varied from 10 to 190 patients with variable assessment time points across chemotherapy regimens.

**Table 1:** Clinical studies of Serum NfL biomarker and its relationship with CIPN symptoms

Author	Cancer type	Chemotherapy drug	Subjects evaluated (n)	CIPN Grading	Assessment time points	NfL pg/mL findings	Prediction
Adra et al. 2024 [17]	Breast cancer	Epirubicin, cyclophosphamide and paclitaxel	10	NCI CTCAE v5 EORTC QLQ-CIPN-20	Baseline, week 3 and post treatment	Baseline median 15.3 pg/mL, wk3 348 pg/mL, post treatment sNfL 49.6 pg/mL	sNfL increases after paclitaxel. High-grade CIPN had significantly higher NfL concentrations
Burgess et al. 2022[32]	Non-small-cell lung cancer	Paclitaxel and carboplatin	88	NCI CTCAE	Screening day 15 prior to treatment, day 1, day 21, and day 126 post start of treatment, 2 years	sNfL levels at day 21 was elevated to both screening tests and day 1. sNfL was significantly raised at day 21 compared to matched baseline scores	sNfL change from first week of therapy predicted subsequent grade 2 and 3 CIPN
Cavaletti et. al. 2023 [27]	Breast cancer	Paclitaxel	49	EORTC-QLQ CIPN20 TNSc	Baseline, 1 month and 3 months posts	Not stated	sNfL concentration was strongly correlated with cumulative dose of chemotherapy
Cebulla et al. 2023 [36]	Multiple myeloma	Bortezomib	70	Neurological examination QST NCS	Variable – during treatment and post treatment	Ongoing treatment: 93.4 pg/mL; post-treatment: 45.3 pg/mL	sNfL concentrations higher in ongoing treatment group and correlated with NCS
Huehnchen et al. 2022 [28]	Breast and ovarian cancer	Paclitaxel	31	TNSr	Baseline and post treatment	Post treatment NfL 60.3 ± 50.4 pg/mL	sNfL > 36 pg/mL associated with greater probability of CIPN post treatment
Karteri et al. 2021 [29]	Breast cancer	Paclitaxel	59	TNSc 44% of women developed grade 2–3 CIPN at week 12	Baseline, week 2, 3 and end of treatment	Baseline 15.3 ± 13.3 pg/mL	Age > 50 years and sNfL > 85 pg/mL at week 3 associated with grade 2–3 CIPN at week 12
Kim et al. 2020 [33]	Colorectal cancer	Oxaliplatin	34	NCI CTCAE	Baseline, 3 and 6 months	Baseline median 12.7 pg/mL, 3 months 22.3 pg/mL and 6 months 115 pg/mL	sNfL > 195 pg/mL at 6 months predicted grade 3 CIPN
Kim et al. 2022 [34]	Gynaecological cancer	Paclitaxel	48	NCI CTCAE	Pre surgery, baseline cycles 2,4, and 6 months	Baseline 65.3–98.9 pg/mL 2nd cycle 103.9–225.8 pg/mL	sNfL > 124 pg/mL after cycle 2 grade 3 CIPN
Mortensen et al. 2022 [35]	Ovarian cancer	Paclitaxel	190	NCI CTCAE	Baseline and at cycle 2 to 6	Baseline median 23.6–27.2 pg/mL, cycle 1 59.9–121.1 pg/mL	sNfL > 150 pg/mL after first cycle had increased risk of CIPN
Velasco et al. 2023 [30]	Breast cancer	Paclitaxel	22	NCS	Baseline, and after 12 cycles (12 weeks)	Baseline 18.50 ± 12.88 pg/mL 12 weeks 255.80 ± 194.16 pg/mL	Increase in sNfL concentrations directly correlated with reduction in NCS recordings
Velasco et al. 2024 [31]	Breast, colorectal and pancreatic cancer	Paclitaxel, brentuximab vedotin, and oxaliplatin	82	TNSc NCI CTCAE NCS	Baseline, after completion of treatment at 3 months and between 6 and 12 months post treatment	Baseline sNfL paclitaxel 16.20 ± 13.62 pg/mL Baseline for oxaliplatin 13.64 ± 6.35 pg/mL	Patients receiving Taxanes had significantly greater and earlier changes in sNfL concentrations compared to those on other drugs and this was associated with greater CIPN severity

*NCI CTCAE* national cancer institute common terminology criteria for adverse events, *EORTC QLQ-CIPN 20* European organization for the research of cancer quality of life chemotherapy peripheral neuropathy 20, *NCS* nerve conduction studies, *sNfL* serum neurofilament light chain, *TNSc* total neuropathy score clinical version, *TNSr* total neuropathy score reduced version

### What steps are needed to validate that a promising sNfL biomarker can predict CIPN clinical outcomes*?*

There is evidence from clinical studies identified in the review (Table 1) that elevated sNfL levels are correlated with cumulative dose of chemotherapy [27], reduced nerve conduction [30], and increasing severity of CIPN symptoms over treatment [17, 35]. An elevated sNfL at week three of paclitaxel chemotherapy predicted grade 2–3 CIPN toxicity at week 12 [29]. Additional studies also suggest that patients who have higher concentrations of sNfL after the first cycle of paclitaxel are at increased risk of severe CIPN post treatment [28, 32, 34]. Drugs such as oxaliplatin show differing patterns of sNfL expression and are associated with later CIPN symptoms [31, 33]. This may be due to the “coasting” effect of the drug causing delayed peripheral neurotoxicity. Coasting is the continued worsening of neurotoxicity for several months after stopping the treatment, particularly with platinum-based compounds because of chemotherapy deposition in dorsal root ganglion neurons [14, 37]. However, the road to clinical implementation is quite long due to the current paucity of validation studies demonstrating that measuring sNfL concentration proactively will have an impact on clinical outcomes. Among the limitations of the previous studies are (a) most paclitaxel studies over other chemotherapy types pose the bias that patterns and predictive value may be different for different agents and (b) trial design should be better tailored taking into consideration timepoint selection, assessment of baseline sNfL, and impact of comorbidities. It could be suggested that for an appropriate clinical study in the CIPN field, recommendations from Gewandter et al. [38] should be taken into account and tailored to the specificity of a biomarker trial implementation as previously described for other neurological diseases [39, 40]. Further, a future biomarker may have regimen-specific “cut off” values that indicate emerging CIPN. Validation of sNfL as a predictive biomarker (early increases predicting later CIPN) will require large, prospective validation studies with pre-specified thresholds and adjustment for confounders (e.g. age, comorbidities potentially causing peripheral neuropathies such as diabetes, endocrine dysfunction, preexisting neuropathy due to another cause, and baseline sNfL); most notably, sNfL should be assessed considering renal function to avoid confounders [41]. Furthermore, sNfL kinetics should be carefully pondered since it is partially unknown [42]: quite recently, it was described in central nervous system related conditions [43], and therefore, it will be pivotal to perfectly match any future study with an appropriate and well-matched control population. Notably, considering, as just noted, that also central nervous system conditions can cause sNfL increase, an accurate evaluation of overlapping/preexisting central nervous system condition would be appropriate.

Group participants discussed the need for large prospective clinical trials or registries with sNfL measured at baseline and early in treatment to validate that sNfL is predictive of later CIPN severity at the end of chemotherapy or post treatment. Existing cohorts of patients enrolled in current studies, for example, SWOG S1714 [44], should be used to validate sNfL and other potentially predictive biomarkers because CIPN is being carefully phenotyped longitudinally using validated subjective patient-reported outcome measures and objective neurologic examination, and serum is being banked. Future studies need to explore whether sNfL predictive validity varies by neurotoxic drug or cancer type. These studies should consider the optimal clinical endpoint with which to validate a CIPN biomarker. CIPN measurement still poses some clinimetric challenges; in this regard, while clinician grading scales such as the NCI-CTCAE have been hard-wired into clinical practice to quantify CIPN, these scales are not reliable or sensitive [45]. As an alternative, a combination of both patient-reported outcome measures, such as EORTC QLQ-CIPN20 and/or FACT/GOG-Ntx, and reliable, sensitive, and responsive objective measures, such as reduced variants of the Total Neuropathy Score©, and functional measures (e.g. Balance, Timed Up and Go, and Peg Board Tests) should be used in sNfL validation studies.

Based on our review of the literature and workgroup discussions, we recommend that future research addresses the following considerations, which are described in Table 2.

1. Test sNfL to predict patient CIPN outcomes via large prospective, longitudinal trials.
2. Initial sNfL validation studies should include sNfL, and comprehensive subjective and objective assessments longitudinally, collected at baseline, 1–2 cycles, mid-treatment, end of treatment, and 3, 6, and 12 months post chemotherapy completion to uncover cut-points and the best time points for prediction, with emphasis on sNfL’s change from baseline to each subsequent timepoint.
3. Test a combination of CIPN biomarkers alongside other clinical or physiological measures to validate one or more CIPN biomarkers for use in a predictive model.
4. Biomarkers of CIPN should be agnostic to cancer type but specific to the chemotherapy treatment regimen.
5. Identify chemotherapy drug-specific cut-points that indicate emerging CIPN for all neurotoxic drug types (e.g. taxanes, platinum, and vinca alkaloids).
6. When possible, leverage existing cohorts from prospective studies that have incorporated rigorous CIPN phenotyping and serum collection/banking to validate sNfL.
7. CIPN measures for use in future sNfL validation studies should include the EORTC QLQ-CIPN20, variants of the Total Neuropathy Score (e.g. TNSc© or TNSn©) plus functional measures.

**Table 2:** Priorities identified from the CIPN biomarker workshop and explanation

**1**. **What steps are needed to validate that a promising sNfL** **biomarker can predict CIPN clinical outcomes** **Priorities for Research**	**Explanation from group 1 participants**
1. Test sNfL to predict patient CIPN outcomes with large prospective trials 2. Select combination of CIPN biomarkers with other clinical or physiological biomarkers 3. Biomarkers of toxicity should be agnostic to cancer type 4. Drug type matters with biomarker and needs to be sensitive to oxaliplatin and vincristine CIPN affects 5. Existing cohorts from prospective studies (such as SWOG cohort should be used) 6. sNfL, CIPN 20, TNSn (pinprick and vibration sensibility) plus balance measures	• Large prospective trials or registries with sNfL measured at baseline and early in treatment to validate that it is predictive of clinical CIPN later/at the end of or post-treatment • Consider whether sNfL should be combined with other clinical or physiological biomarkers, or used by itself • Biomarkers of toxicity should be agnostic to tumour type (i.e. breast vs. ovarian cancer), but may be specific to agent (i.e. paclitaxel vs. oxaliplatin) • Studies should therefore be conducted within drug/class, but across disease states • Existing cohorts from prospective clinical studies (e.g. SWOG S1714) should be used to validate sNfL and other predictive biomarkers • Analyses should carefully consider the optimal clinical endpoint with which to validate a CIPN biomarker. This may be clinician- (NCI CTCAE) or patient- (EORTC QLQ-CIPN20) assessed CIPN, or objective measures including vibration sensitivity or balance
**2. What evidence is needed to demonstrate that sNfL-guided treatment decisions will improve CIPN clinical outcome** **Priorities for evidence of biomarker data linked to clinical outcomes**	**Explanation from group 2 participants**
1. Identify the minimum, core data set through which to evaluate clinical relevance of the proposed innovation in CIPN care 2. The core dataset must substantiate value for the survivor, the clinician, and society at large, as well as address economic impact of the proposed innovation 3. The core dataset of clinically relevant outcomes should be standardised for international deployment to measure effect on: ▪ Recurrence and mortality ▪ Morbidity, meaning symptoms including medical problems caused by treatment: a. *Subjective health*: PROs, e.g. EORTC QLQ-CIPN20, PRO-CTCAE b. *Functional limitations:* e.g. TNS, neuromotor function, dual task function c. *Disease control:* as discussed in Sect. 3	• Phase III studies of superiority are required to determine whether biomarker-guided treatment decisions are better than current usual care. While design of such Phase III study is well established, in terms of multinational site selection and human subjects’ protections, guidance is required to define the outcomes that would substantiate clinical relevance of biomarker-guided treatment decisions • Ideally, Phase III study, conducted across different countries, would apply the same battery of outcome measures, translated to the language spoken per country. Outcomes should include mortality (i.e. do survivors live longer when biomarker-guided treatment decision-making is applied) and morbidity (i.e. are clinically relevant measures of health and wellness improved for survivors who use biomarker-guided treatment decision-making). If superiority of biomarker-guided treatment decisions over usual care is established, then evaluation of the economic implications of biomarker-guided treatment decisions must be considered, including potential cost savings from reduced CIPN-related complications and enhanced decision-making around chemotherapy dosing • We should focus on defining the minimum, core dataset that we can currently recommend for evaluation of the clinical relevance of the proposed innovation. As the state of the science advances regarding accurate, sensitive, and specific measurement of CIPN symptoms, these outcomes should be updated
**3. What are the barriers to and facilitators of sNfL biomarker collection implementation into clinical practice?**	**Group 3 participants**
Barriers	Facilitators
1. Limited evidence of efficacy and validity of sNfL 2. Resource limitations: Limited time & money, extra testing, inequitable resources 3. Technology: High costs, long processing time 4. Variability: Different clinical settings, variable clinical presentations 5. Rationale: lack of patient and clinician understanding of rationale for testing; patient preference 6. Lack of collaborative partner 7. Lack of “gold standard” to benchmark 8. Lack of agreement on cut-offs and threshold for false positivity	• Need for better assessment: Pressing clinical and patient-identified need • Emerging evidence: promising data from pre-clinical, preliminary sNfL studies and small-scale interventional trials • Emerging technology development • Infrastructure: Blood tests are routinely undertaken in cancer care • Other non-invasive testing including digital biomarkers • Emerging applications: Potential acceleration of drug approval/intervention • Enables greater understanding of mechanisms

*CIPN* chemotherapy induced peripheral neurotoxicity, *CTCAE* common toxicity criteria adverse event scale, *EORTC QLQ-CIPN20* European organisation for research and treatment of cancer quality of life questionnaire chemotherapy induced neuropathy 20, *sNfL* serum neurofilament light chain, *PRO* patient reported outcome, *PRO-CTCAE* patient reported outcome CTCAE scale, *TNS* total neuropathy score

### What evidence is needed to demonstrate that sNfL-guided treatment decisions will improve CIPN clinical outcome?

The group discussed which outcomes would be sufficient and clinically meaningful to demonstrate benefit of biomarker-guided treatment decision-making. Dorsey et al. [12] reported that clinical trials of CIPN are needed to establish “parameters that guide the evaluation of clinically meaningful effects”. A consistent limitation noted within the published literature is that there is no standardised method to define CIPN. We sought in the workshop to clarify these parameters. For example, in a hypothetical Phase III trial of standard of care *vs.* biomarker-guided treatment decisions, the group discussed that it would be necessary to assess benefit across three domains: reduced patient-reported symptoms, sustained functional improvements, and disease control (i.e. cancer disease-free survival). The hypothetical Phase III trial should lead to an analysis of economic feasibility regarding implementing biomarker-guided treatment decisions, tailored by country to consider nation-specific health care policies.

To demonstrate clinical relevance, a prospective Phase III study would need to show that a specific biomarker (sNfL) can improve patient outcomes when incorporated in treatment decision-making at a specific point in the chemotherapy treatment cycle. More specifically, biomarkers could be tested at least in two different settings: (a) to drive clinical management and (b) to tailor intervention studies. In the former, sNfL should be tested early during chemotherapy (e.g. after 1–2 cycles) to eventually switch to a non-neurotoxic alternative or dose reduce, an option—dose reduction—that was recently demonstrated as a feasible approach with appropriate tailoring in a population of children/adolescents/young adults affected by Hodgkin lymphoma [46] and that was also already applied successfully in adults affected by multiple myeloma [47]. However, the main issue here is that we do not have a validated cut-off for sNfL to predict the neurological outcome at the end of chemotherapy, even though there are some preliminary studies [29]. Thus, research is still needed on large cohorts to ascertain this aspect. In perspective, in order to define sNfL relevant threshold values/increments, patients would be randomly assigned to usual care alone or to biomarker-guided care (e.g. chemotherapy dose adjustments or other interventions as they become available) supplemented by biomarker analysis at key clinical decision-making timepoints, such as after the first cycle of chemotherapy. The study would test the hypothesis that biomarker-guided clinical decision-making is superior to usual care regarding morbidity (i.e. less severe or persistent CIPN presentation), without negatively impacting disease control or mortality. Perhaps, enrichment designs should be considered whereby patients with high sNfL levels after cycle 1 or 2, but no clinical symptoms, would be randomized to adjusted chemotherapy dosing versus continuation of standard of care.

Moreover, once more robust data on sNfL normal values/relevant increments will be available, as already mentioned, preventive clinical trials would greatly benefit from introducing biomarkers as a pivotal element in study design. For example, sNfL would be measured early during chemotherapy (e.g. 1–2 cycles into treatment) and only patients with a relevant increase and, therefore, at actual risk of developing CIPN would be enrolled, avoiding a dilution effect by having study participants who will not develop CIPN at all for their individual characteristics.

Ideally, this Phase III trial would test the use of biomarker-guided treatment decision-making in conjunction with specific interventions known to improve CIPN. Because the observed change in biomarker concentration could trigger well-evidenced clinical interventions, the interventions to be triggered in the decision-making process should be defined in advance. This is important to increase the chance of detecting clinically relevant change with biomarker-guided treatment decision-making. For instance, dose reduction is believed to limit severity and persistence of CIPN presentation; therefore, dose reduction is a candidate intervention that would be triggered by specific changes in the biomarker of interest (e.g. NfL alone or in combination with other predictive biomarkers). However, longitudinal descriptive studies that show the patterns of CIPN and concurrent biomarker changes are needed first, with statistical control for neurotoxic drug dose reductions. This type of data could show that an early or extremely high biomarker spike experienced during chemotherapy is predictive of worse long-term outcomes. These data could then support a Phase III trial of biomarker-informed treatment decisions, particularly in patient populations that will be cured and are at risk for late effects and chronic sequelae.

Another way to evaluate biomarker value is to show that values drop in response to effective CIPN interventions, such as duloxetine which was suggested as a symptomatic treatment for painful CIPN [48], exercise interventions [49], or other interventions as they emerge. These types of studies can illustrate that a valid CIPN biomarker could be used as primary outcome measures in future intervention studies. Because intervention outcomes are moderated by adherence, the clinical trial will be required to document adherence to the interventional activity and incorporate this variable within the statistical analysis.

Based on our review of the literature and workgroup discussions, we recommend the following as detailed in Table 2:

1. Refining study design by identifying the minimum, core data set through which to evaluate clinical relevance of the proposed sNfL biomarker innovation to improve clinical outcomes.
2. Defining NfL relevant threshold values; the core dataset must substantiate value for the patient, the clinician, and society at large, as well as address the economic impact of sNfL biomarker implementation.
3. The core dataset of clinically relevant outcomes in future studies should be standardized for international deployment to measure effects on:
  Cancer recurrence and mortality
  Chemotherapy-induced treatment administration per standard-of-care recommendations
  Morbidity, meaning symptoms including medical problems caused by treatment:
    a *Subjective symptoms*: Patient-Reported Outcomes (e.g. EORTC CIPN20, PRO-CTCAE) [5, 50]
    b *Brief neurological assessment*: TNSc© or TNSn© [51, 52]
    c *Functional limitations:* Jamar Grooved Pegboard test, Timed Up and Go, Balance [53]

### What are the barriers to and facilitators of sNfL biomarker collection implementation into clinical practice?

The barriers and facilitators of a CIPN biomarker clinical implementation were discussed in detail and are listed in Table 2. Chiefly, the lack of validated evidence of biomarker predictive validity, such as sNfL, has limited implementation to date. As a facilitator, there is already substantial infrastructure to support the use of blood-based biomarkers in routine oncology practice; for example, tumour biomarkers are used to monitor disease outcomes. There is also promising preliminary data surrounding biomarker clinical validity from both patient and clinician perspectives [54, 55]. As a barrier to clinical implementation, on top of all issues described in depth in Table 2, the main one is that the current high costs and slow turn-around time for sNfL testing may preclude implementation without technological advances to address these barriers so that there is point-of-care testing that can inform just-in-time decision-making regarding chemotherapy dosage adjustments or use of other interventions when available.

## Discussion

Workshop participants agreed that there is currently not enough evidence of ‘clinical utility’ for implementation, i.e. evidence that using the sNfL biomarker improves clinical outcomes for patients with cancer. There was consensus that sNfL might not be sufficient as a predictive tool of CIPN and that longitudinal collection of a panel of biomarkers and other validated CIPN assessments would be needed to demonstrate a predictive relationship between early biomarker values and CIPN-associated outcomes. The clinical studies in the review identified the wide variance in individual sNfL levels and the differentiation between types of chemotherapy drugs and between cohorts. This variance makes sNfL difficult to use currently in treatment decision-making. There was consensus that larger prospective clinical studies are therefore needed in well-characterized cohorts of cancer patients to be able to identify the positive and negative predictive value of a CIPN biomarker. If superiority of biomarker-guided treatment decisions over usual care is established, then evaluation of the economic implications of biomarker-guided treatment decisions must be considered, including potential cost savings from reduced CIPN-related complications and enhanced decision-making around chemotherapy dosing.

There are multiple ways to assess CIPN (e.g. physician-based or patient-based outcome measures). Some of them such as the clinician graded CTCAE assessments are not sensitive or reliable [51], whereas neurologic exams and nerve conduction studies sometimes are not feasible for use in busy practice settings. A biomarker blood test, once validated, if results were immediately available, would be much more clinically useful for guiding clinical decisions at the point-of-care. Thus, the need for researchers to move forward with further research in order to then implement biomarkers in the CIPN field.

Differentiating early patients at high risk of developing severe CIPN is one of the challenges of clinical practice as currently there are limited interventions for resolving CIPN that will be acceptable to patients and clinicians alike and that do not reduce treatment efficacy (i.e. dose reduction/discontinuation). In the future, these challenges could be addressed by implementing biomarker monitoring at each chemotherapy treatment visit to detect high-risk patients who should be considered for early CIPN-mitigating interventions. In the current lack of a sound treatment to prevent CIPN, they could drive the decision to dose reduce or shift to a non-neurotoxic alternative if available, but in the future, as soon as CIPN preventive treatments will be hopefully available, biomarker testing could be used to inform the decision to start the neuroprotective treatment.

## Conclusion

Severe CIPN is a significant clinical problem that affects patients’ long-term quality of life and drives clinical decisions regarding chemotherapy dosing. To identify a predictive biomarker of severe and chronic CIPN that can be used to guide clinical decision-making, we need large prospective longitudinal studies that demonstrate the value of sNfL as a biomarker. Clinical studies show promise but highlight significant variability in sNfL based on the measurement timepoints and differing chemotherapy drugs. Further, given that there are multiple mechanisms of chemotherapy neurotoxicity, it will be important to test mechanism-specific biomarkers; as such, more than one biomarker may be needed beyond sNfL to detect the impact of chemotherapy. For clinical decision-making, a high level of positive predictive validity is needed to justify changing chemotherapy treatment regimens because the clinician and patient must balance the risk of CIPN against potentially compromised cancer outcomes. Therefore, future studies are needed to test biomarker sensitivity and predictive validity, define standards for when and how frequently to test, and identify chemotherapy drug-specific cut points that should trigger provision of anticipatory CIPN management, including rehabilitation. Studies are also needed that show that NfL-informed care will improve outcomes. Lastly, point-of-care testing technology will be needed before NfL can become a mainstay tool to inform just-in-time clinical decision-making. Despite the numerous barriers, published literature and opinions from expert workgroup participants support the potential of sNfL to inform clinical practice decisions and ultimately improve many patient outcomes. Thus, future research in this area is warranted before sNfL would likely be incorporated as a secondary outcome in CIPN neuroprotection trials and/or used to inform clinical practice.

## Data Availability

No datasets were generated or analysed during the current study.
